# Motor Intention Drives Corticospinal Facilitation during Interception Planning

**DOI:** 10.64898/2026.01.13.699289

**Published:** 2026-01-14

**Authors:** Justin R. McCurdy, Brendan Jarvis, Emma Whitman, Krithi Ariga, Asher Khan, Deborah A. Barany

**Affiliations:** 1 Department of Kinesiology, University of Georgia, Athens, GA 30602; 2 Neuroscience Program, University of Georgia, Athens, GA 30602; 3 Department of Interdisciplinary Biomedical Sciences, University of Georgia, Athens, GA 30602; 4 Regenerative Bioscience Center, University of Georgia, Athens, GA 30602

**Keywords:** corticospinal excitability, motor preparation, eye movements, interception planning, visuomotor control, transcranial magnetic stimulation

## Abstract

Intercepting a moving target requires integrating visual motion information with motor preparation, yet how action intention and gaze strategy jointly shape corticospinal excitability during interception planning remains unclear. We applied single-pulse transcranial magnetic stimulation over primary motor cortex to probe corticospinal excitability while participants either prepared to intercept a moving target or passively viewed its motion. Targets moved at one of two speeds, and participants judged target speed relative to a reference after each trial. Across blocks, participants were instructed to either smoothly pursue the target or fixate their eyes on the interception zone. Motor-evoked potentials were elicited at baseline, after task instruction, early during target motion, or shortly before target arrival. Task goal and gaze strategy interacted to influence both perceptual judgments and corticospinal excitability. Passive viewing led to overestimation of target speed and was associated with sustained suppression of corticospinal excitability, particularly during fixation. In contrast, preparing to intercept produced a robust facilitation of corticospinal excitability immediately before movement onset, regardless of gaze strategy. Smooth pursuit improved perceptual accuracy and reduced interception timing error compared with fixation but did not independently influence corticospinal facilitation. These findings demonstrate that the intention to act is the primary determinant of the transition from corticospinal suppression to facilitation during interception planning, whereas eye movements modulate perceptual estimates and support behavioral performance.

## Introduction

Intercepting a moving object relies on the brain’s ability to rapidly predict a target’s trajectory and time an appropriate motor response. Visual motion cues during interception shape perceptual judgments and prime the motor system for action, supporting both spatial and temporal accuracy under variable dynamics and environmental uncertainty^1–3^. Because interception requires integrating visual motion signals with motor commands, these cues may not only inform perceptual judgments but also provide predictive information about target kinematics^4,5^. In turn, engaging the motor system can enhance motion perception^6^, suggesting a bidirectional link between visual motion perception and interception planning.

Additionally, gaze behavior plays a crucial role in visually guided interception^7^. Smooth pursuit eye movements (SPEM) stabilize foveal input by matching ocular velocity to target speed and providing continuous, high-resolution feedback^8,9^. In contrast, endpoint fixation holds gaze on an anticipated interception zone, relying on peripheral cues and internal predictive models to estimate target arrival. In scenarios with predictable motion, fixating on an interception location can be advantageous, whereas when motion is less predictable, SPEM tends to enhance interception performance^7,10,11^. Taken together, these findings indicate that interception performance emerges from the coordinated interactions of motor preparation, visual motion processing, and gaze behavior.

In humans, transcranial magnetic stimulation (TMS) has been used to non-invasively probe corticospinal excitability (CSE) across movement stages. Preparatory CSE typically exhibits a biphasic pattern, with an early inhibitory phase followed by a facilitation peak immediately before movement onset^12–15^. Previous research has identified that the extent of CSE modulation scales with task demands on action preparation, such as switching between plans to initiate, withhold, or cancel a response^16–21^. CSE is also affected by the complexity of the motor plan^22^ and modulated by visual information, such as uncertainty of motion cues during preparation^23,24^. Therefore, modulation of CSE during interception planning may reflect contributions from both motor intention and the integration of task-relevant visual cues.

Even in the absence of overt movement preparation of the targeted effector, visual input and task demands can modulate CSE. Classic studies using action observation paradigms have found that CSE increases when watching the actions of others^25–27^. This facilitation is enhanced when the visual stimuli are behaviorally relevant^28^, paired with imagery^29,30^, or specific to motion kinematics^31^. The type of visual feedback of one’s own actions (e.g., movement of the opposite limb) can also modulate CSE in an effector-specific pattern^32,33^. These results support the hypothesis that CSE reflects the priming of the motor system for simulation of goal-directed action^34^.

Some of the visual-related modulation of CSE in the intrinsic hand muscles during action observation may be attributed to differences in eye movements across conditions. For example, enhanced CSE is associated with more fixations on goal-relevant information^28^. Smooth pursuit eye movements can influence motor readiness by modulating long-latency reflexes in the lower limb during stance, with reflex responses to perturbations significantly larger during pursuit than during fixation^35^. In addition, smooth pursuit eye movements in the absence of visual feedback globally suppresses CSE of the hand muscles^36^, indicating that oculomotor commands can directly influence excitability. These findings suggest that gaze behavior is not merely a passive consequence of visual processing, but an active contributor to motor preparatory states^37^.

Our previous work found evidence that CSE is facilitated while preparing to intercept faster moving targets, demonstrating that visual motion kinematics can influence preparatory dynamics^5^. However, the observed differences between intercepting fast and slow targets could arise from multiple sources, including differences in visual input, differences in imposed task demands, and differences in gaze strategy. In the present study, we aimed to disentangle the respective contributions of these different factors to changes in corticospinal output during timed interceptions. We measured TMS-evoked motor cortical potentials (MEP) during interception planning and passive observation of moving targets. Across blocks, we manipulated eye movement strategy, contrasting smooth pursuit with endpoint fixation, to examine whether gaze behavior modulates task-specific excitability.

We found late facilitation exclusively during active interception planning, with limited modulation by eye movement strategy. This pattern demonstrates that motor intention, rather than sensory input alone, drives the transition from suppression to facilitation in corticospinal circuits. By revealing this intent-dependent mechanism, our findings clarify how the motor system integrates predictive visual information with preparatory commands to support accurate interception.

## Methods

### Participants

Twenty healthy volunteers (10 M, 23.7 ± 4.3 years) took part in the study. All participants were right-handed, had normal or corrected-to-normal vision, reported no history of neurological disorders, and had no contraindications to TMS. Written informed consent was obtained from all participants, and the study was approved by the Institutional Review Board at the University of Georgia, in accordance with ethical guidelines for human research.

### Experimental Setup

Participants were seated in front of the task monitor (1920 × 1080 resolution, 60 Hz refresh rate) at a desk with their right hand resting on a digitizing trackpad (WACOM Intuous Pro Large). The experiment and stimulus presentation were controlled using PsychoPy 3 (Peirce et al. 2019). The trackpad was positioned so that participants could rest both arms comfortably on the desk, while placing their chin in a custom stand approximately 58 cm from the center of the screen. Participants wore earplugs for the duration of the study and a sleeve over their index finger to enhance trackpad sensitivity. Surface electromyography (EMG) activity of the FDI and ADM muscles of the participant’s right hand were recorded using a Delsys Trigno wireless system (Delsys Inc., Boston, MA, USA). EMG signals were amplified 1000x, bandpass filtered (20–450 Hz), and sampled at 5000 Hz through a data acquisition board (Micro 1401-4, Cambridge Electronic Design, Cambridge, UK) and CED Signal software. Monocular eye position was recorded at 500 Hz and monitored in real-time using a high-precision, video-based eye-tracking system (Eyelink 1000 Plus, SR Research, Ottawa, ON, Canada).

### Transcranial Magnetic Stimulation

TMS was applied through a 70-mm, figure-of-eight coil driven by a DuoMAG MP magnetic stimulator (Deymed Diagnostics sro, Hronov, Czech Republic). MEPs were elicited from the right FDI by applying TMS over the hand area of the left M1. The coil was placed tangentially on the scalp with the handle oriented towards the occiput and laterally from the midline at a 45° angle. Before each of the experimental sessions, the FDI motor hotspot (i.e., the optimal location for eliciting MEPs in the target muscle) was identified on each participant by eliciting TMS every 4 s and systemically repositioning the coil. At the beginning of each experimental session, a model of the brain was scaled to the participant’s cranial dimensions using a neuronavigational tracking system (BrainSight, Rogue Research, Montreal, Canada) to optimize and replicate coil position throughout the session. Resting motor threshold (rMT) was defined as the minimal TMS intensity required to evoke peak-to-peak MEP amplitudes of ~50 uV in the target muscle for 5 of 10 consecutive trials ^38^. The average rMT for this experiment was 41.1% (± 4.7) of the maximum stimulator output. Stimulator intensity during the task blocks was set to 115% of the participant’s rMT. TMS timings were controlled by sending TTL pulses from the task computer to the stimulator via Signal software (Cambridge Electronic Design, Cambridge, UK).

### Experimental Design and Procedure

**Figure 1.**
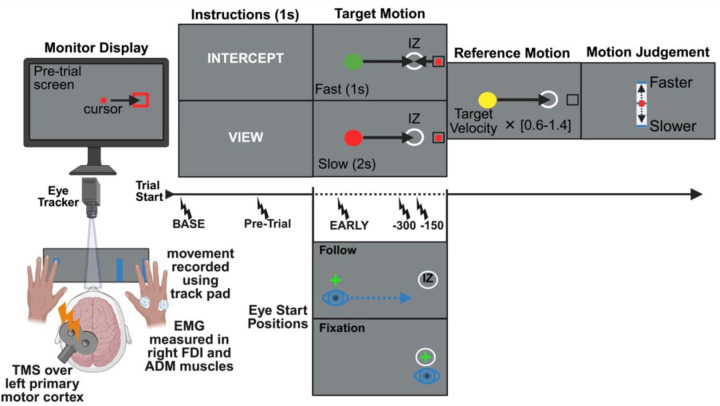
Interception task design. Participants completed an interception task requiring them to estimate the time a moving target reached a predefined interception zone (IZ) while following assigned eye movement strategies (fixate on the interception zone or follow the moving target). At the beginning of each trial, participants were instructed to either intercept the target with a swipe gesture or passively view its motion. The target moved at one of two constant velocities (Fast or Slow), and after each trial, a reference object appeared, requiring participants to judge whether it was faster or slower than the original target. Eye tracking recorded gaze position, while TMS was applied over left M1 at baseline or one of three timepoints during movement preparation (Early: 100ms after target onset, −300: 300ms before target-IZ overlap, −150: 150ms before target-IZ overlap). EMG activity was measured from the FDI and ADM muscles of the right hand to analyze movement initiation and corticospinal excitability.

Participants performed a combined speed discrimination and interception task in which they judged the speed of a moving target while simultaneously intercepting it in a predefined interception zone or passively viewing it (Fig. 1). On separate experimental days, participants employed different gaze strategies: they were instructed to either smoothly track the target with their gaze (“Follow”) or maintain fixation on the interception zone (“Fixate”) until the target arrived.

In the “pre-trial” phase, participants first moved their right index finger on the touchpad to move an on-screen cursor (red rectangle, 0.5 × 0.5 cm) into a fixed start position. Once the cursor was in the start position for 100 ms, participants were cued to either intercept the moving target by abducting their right index finger on a touchpad (“Intercept”) or simply view its motion without acting (“View”). After 1 s, the instruction cue would disappear, and participants would move their eyes to the appropriate eye start location: either the target’s start position for the Follow session or the interception zone (circle, 0.85 cm radius) for the Fixation session, denoted by a small green fixation cross that disappeared once gaze was detected for more than 300 ms.

After the pre-trial phase, a green (Intercept trials) or red (View trials) target (circle, 0.7 cm radius) would appear 24 cm to the left of the interception zone. After a brief static delay (300 ms), the target swept across the screen at one of two constant horizontal speeds, 12 cm/s (“slow”) or 24 cm/s (“fast”), until it reached the interception zone. On Intercept trials, participants were instructed to abduct their right index finger to intercept the target as it arrived at the interception zone. On View trials, participants were instructed to keep their hand at rest throughout the trial.

Once the target stopped, participants were instructed to move their eyes back to the start position for that trial. Once gaze position was confirmed (as before), a yellow reference target appeared, remaining static for 300 ms before retracing the same horizontal motion path as the primary target, but at a different speed. The speed was drawn at random from six multipliers of the trial speed (0.6, 0.8, 0.9, 1.1, 1.2, or 1.4), making it slower or faster than the primary target (24 vs. 12 cm/s). After watching the reference target motion, participants indicated whether it moved faster or slower than the primary target by sliding a cursor along a continuous scale and pressing a button using their left hand to submit.

Prior to the main task, participants completed repeated practice blocks consisting solely of interception trials until they achieved at least 80% accuracy—that is, successful interceptions within 90 ms of the target’s arrival at the interception zone. During practice, participants received immediate feedback after each trial, indicating whether their response was “Too Early,” “Too Late,” or “Perfect.” After reaching criterion, participants completed a 48-trial block with unrestricted eye movements, followed by a 48-trial eye-training block. No TMS was applied during these training blocks. Instead, participants were instructed on how to implement the designated eye strategy for that session. Both Intercept and View trials were included, and gaze behavior was continuously monitored to ensure compliance with fixation or smooth pursuit instructions.

To evaluate CSE modulation during preparation, transcranial magnetic stimulation (TMS) was applied on 43 of the 49 trials in each task block under three different timing conditions: (1) a baseline stimulation delivered prior to the instruction window when no stimuli were present, (2) a post-instruction stimulation administered after the response cue but before the target’s appearance, and (3) trial stimulation delivered at one of three specific time points: 200 ms after target onset (Early), 300 ms before target arrival at the interception zone, or 150 ms before target arrival. In the remaining 6 trials, no TMS was applied. The order of the eye movement strategy condition for each experimental session (“Follow” or “Fixate”) was counterbalanced across participants. In each experimental session, participants performed 5 task blocks of 49 trials each. Task instruction (Intercept vs. View), target speed (Fast vs. Slow), and TMS timing (baseline, post-instruction, Early, 300 ms, 150 ms) were pseudo-randomized across trials to minimize anticipation effects. Altogether, there were 15 trials performed per each unique condition.

### Data Analysis

EMG recordings were analyzed offline using the MATLAB VETA toolbox^39^. Interception performance was quantified by movement onset, movement time, and interception timing. Movement onset was identified as the first timepoint EMG activity in the FDI muscle exceeded 3 standard deviations of the mean of the rectified signal for the entire trial epoch and was >0.5 mV, relative to the target’s arrival time. Movement time was defined as the duration from EMG-derived movement onset to the time at which the interception zone was reached. Interception time was defined as the absolute timing error between the time the target and finger position entered the interception zone.

To quantify speed perception, participants’ judgments on the continuous scale were converted to binary responses (reference target faster or slower than the primary target). These binary judgments were fit to a sigmoid psychometric function, from which we extracted the point of subjective equality (PSE) in units of percent speed difference. Negative PSEs indicate that the reference target was judged as slower than the primary target (primary target speed overestimated); positive PSEs indicate the reference target was judged as faster than the primary target (primary target speed underestimated). For Follow eye movements, smooth pursuit eye movements were identified when gaze and object locations remained continuously within a foveal visual radius of ~2° visual angle from the moving target, consistent with established criteria for pursuit detection ^8,9^. Saccades were detected when instantaneous eye velocity exceeded 30°/s or acceleration exceeded 8,000°/s², indicating rapid shifts in eye position^40^. SPEM gain, a measure of SPEM quality, was calculated by dividing gaze angular velocity by target angular velocity over the middle 40% of each target motion period (i.e., we removed the first and last 30% of each trial period before computing SPEM gain). A SPEM gain of 1 indicates ideal target tracking; less than 1 indicates the eye lagged the target; greater than 1 indicates the eye led the target.

CSE was indexed by the TMS-elicited MEP peak-to-peak amplitude. The root mean square (RMS) value of the background EMG activity was calculated for the 300 ms before the TMS artifact. Trials were rejected from analysis when the background RMS was ±2 standard deviations from the participant’s average RMS or when EMG activity preceded the MEP. account for changes in MEP amplitude due to fluctuations in background EMG activity^41^. The peak-to-peak amplitude of the EMG signal was calculated during the 20–50 ms window after TMS (i.e., the normal range at which an MEP is expected to occur). Each participant’s MEP values were normalized to the participant’s average within-task baseline MEP amplitude.

### Statistical Analysis

Statistical analyses were performed in R (v4.2.1+) using the lme4, lmerTest, and emmeans packages, with a two-tailed α level of 0.05. Prior to modeling, each outcome measure was screened for normality (Kolmogorov–Smirnov) and variance homogeneity (Levene’s test). Given our within-subject design, we fit linear mixed-effects models (LMMs) using restricted maximum likelihood (REML). Fixed effects were assessed via Type III ANOVA using Satterthwaite’s approximation for denominator degrees of freedom, and random-effect structures (participant intercepts and by-condition or by-factor slopes) were chosen based on likelihood-ratio tests. Post-hoc pairwise and one-sample comparisons were conducted on estimated marginal means (emmeans) and adjusted for error with the Holm procedure.

Smooth pursuit gain was modeled with Target Speed (Fast vs. Slow) and Task Goal (Intercept vs. View), and their interaction. The model included participant-level random intercepts and random slopes for Target Speed. Perceptual thresholds (PSEs) were modeled with Eye Strategy (Fixation vs. Follow), Task Goal, and Target Speed, plus their interactions, again including participant intercepts and by-strategy slopes. Interception kinematics – movement onset latency, movement time, and normalized interception timing – were each analyzed with Eye Strategy × TMS Timing (NoTMS, Early, −300 ms, −150 ms) and participant-specific intercepts and by-TMS slopes. Corticospinal excitability was examined in two stages: (1) pre-trial instruction effects were assessed with an LMM predicting normalized MEP amplitude at 200 ms post-cue from Eye Strategy and Instruction, covarying baseline EMG (RMS), and followed by Holm-corrected one-sample t-tests against baseline for each Eye × Instruction cell; and (2) in-trial stimulation effects were tested with an LMM including Task Goal, Eye Strategy, and TMS Timing, covarying pre-stimulation EMG (RMS) and modeling by-factor random slopes, with subsequent one-sample t-tests comparing each Task Goal × Eye Strategy × TMS condition to baseline.

## Results

### Targets were perceived as slower when intercepting and when engaging in smooth pursuit

**Figure 2.**
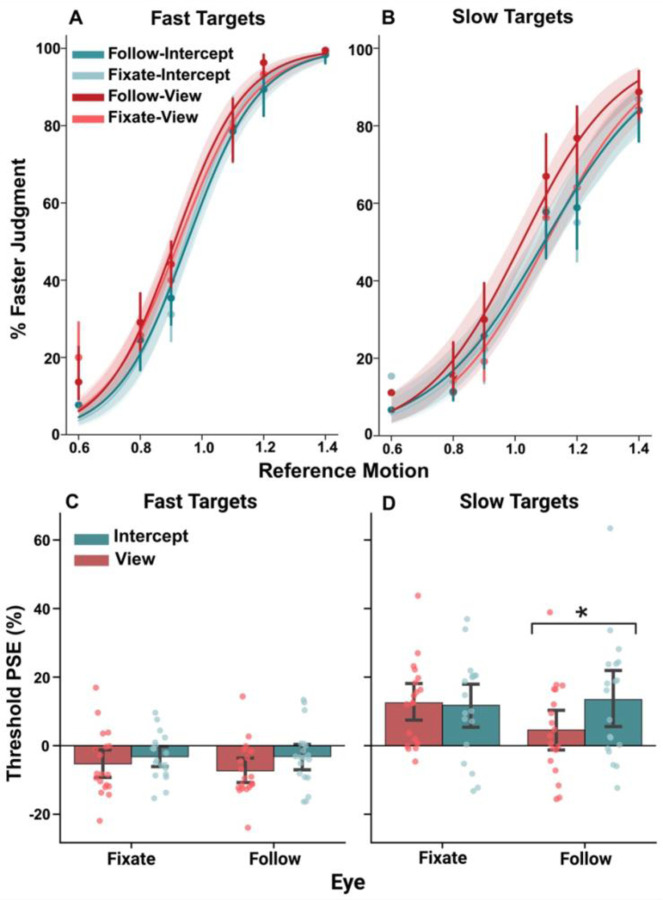
Overestimation of fast target speed and underestimation of slow target speed **(A–B)** Group-average psychometric functions for Fast **(A)** and Slow **(B)** targets under fixation (dashed lines) and smooth pursuit (solid lines) in both View and Intercept conditions. The reference speeds ranged from 0.6 to 1.4 times the primary target speed. **(C–D)** Group-averaged points of subjective equality (PSE) for Fast **(C)** and Slow **(D)** targets, with 95% confidence intervals. *p < .05 between conditions (Holm-corrected).

We first assessed how action intent and eye movement strategy influence perception of the moving target’s speed. Figure 2A,B plots the group averaged psychometric functions, separately for Fast and Slow targets. Visual inspection suggests systematic differences in slope between Fast and Slow target conditions, though this may reflect scaling of the reference stimulus rather than true differences in sensitivity. Figure 2C, D plots mean points of subjective equality (PSE), defined as the percent speed difference at which the second (reference) target was perceived as matching the first (primary) target; positive PSEs indicate underestimation of the primary target speed, whereas negative PSEs indicate overestimation. There was strong main effect of target speed (*F* = 22.81, *p* < 0.001), indicating that participants overestimated the speed of Fast targets (negative PSEs) and underestimated the speed of Slow targets (positive PSEs). The main effect of task goal was also significant (*F* = 7.19, *p* = 0.015), indicating that participants judged the primary target as moving slower when participants planned an interception.

There was no main effect of gaze strategy (*F* = 2.03, *p* = 0.171). However, there was a significant task goal by eye movement strategy interaction (*F* = 5.91, *p* = 0.042). Post-hoc tests revealed that when participants followed the target with their eyes, PSEs were significantly more positive in Intercept trials relative to View trials (*p* < 0.007). In contrast, when participants fixated on the goal, the Intercept-View difference was small and non-significant (*p* = 1.00). This pattern indicates that perceived primary target speed was slowest when participants engaged smooth pursuit eye movements while preparing to intercept the target. Together, these results demonstrate that both action intention and gaze strategy influence perceptual thresholds when interpreting target speed.

### Smooth pursuit eye movements improved interceptive timing

**Figure 3.**
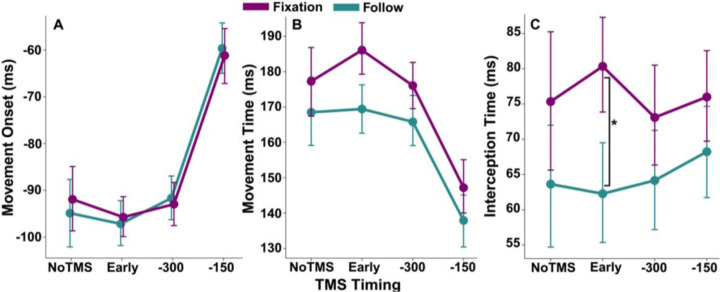
Smooth pursuit eye movements improved interception timing. **(A)** Movement onset relative to target arrival, showing later initiation for −150ms compared to NoTMS. **(B)** Movement time, indicating shortened duration for late simulation (−150). **(C)** Interception time, demonstrating improved accuracy when following the target with gaze. Error bars represent 95% confidence intervals. *p < 0.05 between conditions (Holm-corrected).

As expected, TMS timing significantly impacted movement onset (*F* = 18.47, *p* < 0.001; Fig. 3A). Post-hoc tests showed that TMS delivered 150 ms before target arrival significantly delayed movement relative to NoTMS (*p* < 0.001). Early-trial and −300 ms stimulation did not differ from NoTMS (*p*’s > 0.05). All comparisons with the −150 ms condition revealed significant delays across eye conditions (*p*’s < 0.05), indicating that stimulation closer to movement initiation disrupts anticipatory preparation. Eye movement strategy did not independently affect movement onset (*F* = 0.02 *p* = 0.887), nor did it significantly interact with TMS timing (*F* = 0.18 *p* = 0.912), indicating that movement initiation was primarily modulated by the time of TMS independent of eye movement strategy.

Increases in movement onset at later TMS timepoints were accompanied by decreases in movement time (*F* = 19.13, *p* < 0.001; Fig. 3B). Stimulation at −150 ms led to faster movement times compared to the baseline condition (*p* < 0.001), suggesting compensation for delayed movement onset. There was a trend such that movement times were slower for Fixate movement blocks (*F* = 19.13, *p* = 0.071), with no interaction between TMS timing and eye movement strategy (*F* = 0.88, *p* = 0.452).

Eye movement strategy significantly influenced interception timing (*F* = 7.71, *p* = 0.011; Fig. 3C). Post-hoc tests showed significantly decreased timing error for the Follow eye movement strategy when TMS was applied early in the trial (*p* = 0.008), indicating that tracking the target with smooth pursuit improved interception precision. In contrast, there was no significant effect of TMS time (*F* = 0.66, *p* = 0.580) and no significant interaction between eye movement strategy and TMS time (*F* = 1.39, *p* = 0.243), suggesting that stimulation did not independently enhance or disrupt interception accuracy.

### Speed-dependent smooth pursuit gain during motion perception and interception

**Figure 4.**
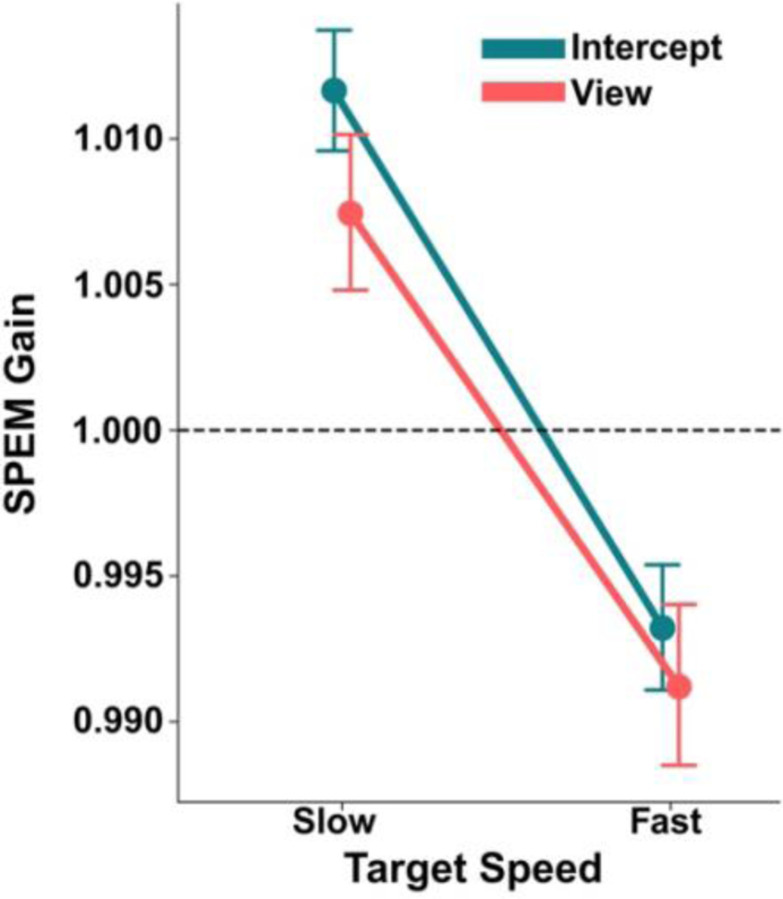
Smooth pursuit eye movement gain similar during interception and passive viewing. Eye movement velocity exceeded target velocity for slow targets and was below target velocity for fast targets for both task goals (Intercept and View). Error bars correspond to 95% confidence interval.

To evaluate the effect of action intent on smooth pursuit quality, we quantified SPEM gain during the Follow eye movement blocks (Fig. 4). There was a main effect of target speed on SPEM gain (*F* = 68.94, *p* < 0.001), indicating that participants’ eye movements tended to lead the more slowly moving target, and lag behind the faster target. SPEM gain tended to be higher in the Intercept condition, though neither the main effect of task goal (Intercept vs. View; *F* = 2.28, *p* = 0.149) nor its interaction with target speed (*F* = 1.49, *p* = 0.222) were significant. This suggests that target speed is the primary driver of pursuit gain, with similar overshooting at slower speeds and lagging at higher speeds for both interception and passive tracking task contexts.

### Late corticospinal facilitation during interception, but not passive viewing of moving targets

**Figure 5.**
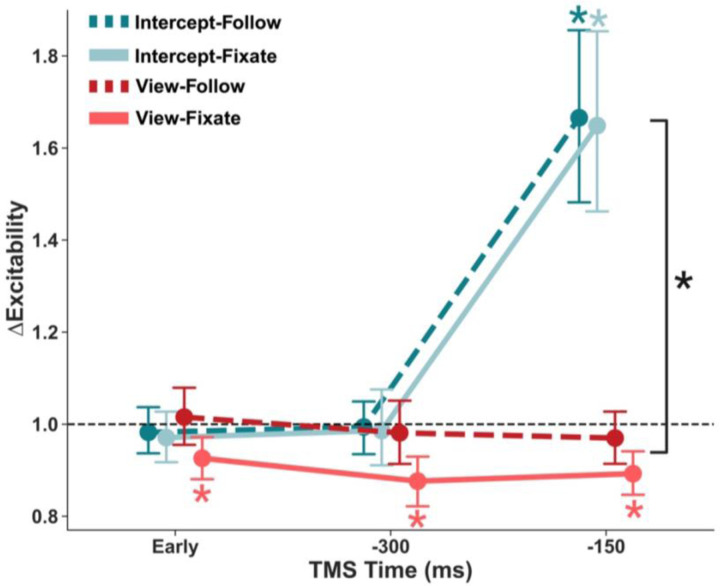
Intent-Driven Modulation of Corticospinal Excitability. Average normalized motor-evoked potentials for the FDI muscle are shown across three TMS time points (Early, −300 ms, and −150 ms) for each combination of eye movement strategy (Follow vs. Fixate) and task goal (Intercept vs. View). CSE increased significantly as target arrival approached the interception zone, with the strongest facilitation at −150 ms during interception trials (Follow-Intercept and Fixate-Intercept). In contrast, CSE remained suppressed during passive viewing trials (Follow-View and Fixate-View), particularly a −150 ms. Error bars represent bootstrapped 95% confidence intervals. *p < 0.05 relative to zero (Holm-corrected).

To address our main question on the relative contributions of motor intent and eye movements on CSE modulation, we calculated changes in FDI MEP amplitudes at the three TMS latencies (Early, −300 ms, −150 ms) for every combination of gaze strategy (Fixate vs. Follow) and task goal (Intercept vs. View) (Fig. 5). There were significant main effects of task goal (Intercept > View; *F* = 7.15, *p* = 0.014) and TMS timing (*F* = 6.16, *p* = 0.005), indicating overall higher CSE when intercepting and when stimulation was closer to the target’s arrival in the interception zone. There was no effect of eye movement strategy (*F* = 0.26, *p* = 0.616). Pre-TMS EMG (RMS) strongly predicted MEP size (*F* = 66.57, *p* < 0.0001), suggesting higher MEP amplitudes when background EMG activity was higher.

A significant interaction between task goal by TMS time (*F* = 105.92, *p* < 0.001) showed CSE modulation depended on the motor intent. Follow-up tests showed that when TMS was applied 150 ms before the target’s arrival, there was clear facilitation when the goal was to intercept, but not during passive viewing *(p* < 0.001). There were no significant differences between Intercept and View trials at the other TMS timepoints (*p’s >* 0.05). To establish deviations from resting baseline, one-sample t-tests were run for each unique condition. Intercept trials at −150 ms exhibited robust facilitation in both gaze conditions (Fixate: *t* = 6.40, *p* < 0.001; Follow: *t* = 6.77, *p* < 0.001), whereas no interception comparisons at the −300 ms or the Early timepoints differed from baseline (all *p’s* > 0.05). In contrast, View-Fixate trials showed consistent suppression at every latency (−150 ms: t = −4.21, p < 0.001; −300 ms: *t* = −4.63, *p* = 0.001; Early: *t* = −3.21, *p* < 0.001), while View-Follow trials remained indistinguishable from baseline at all timings (all *p’s* > 0.05). Together, these results confirm a late, intent-driven up-regulation of corticospinal excitability immediately before movement onset when planning an interception, independent of gaze strategy. In contrast, passive viewing, especially under fixation, induces a timing-independent suppression of motor output.

### Task response instruction induces small changes in corticospinal excitability

**Figure 6.**
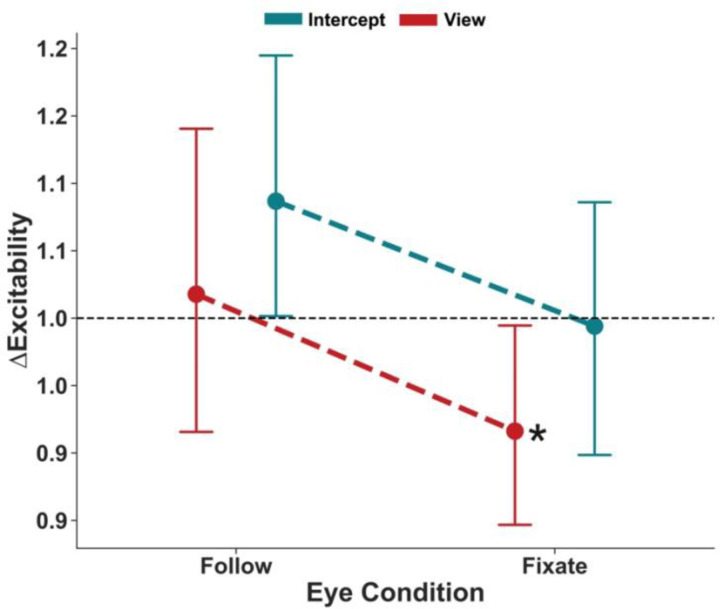
Task Instruction Induces a Modest Suppression During Passive Viewing with Fixation. CSE normalized to baseline for the FDI muscle are shown for each eye movement condition (Follow vs. Fixate) across task instruction conditions (View vs. Intercept). During View trials, there was significantly suppressed excitability relative to baseline during Fixation. There were no significant differences in CSE between eye movement and task instruction conditions. Error bars represent 95% confidence intervals. *p < .05 from baseline (Holm-corrected).

Action intent may influence motor readiness even before active movement preparation begins. To assess this possibility, we applied TMS pulses 200 ms after participants received the task instruction (Intercept or View), but prior to target motion onset (Fig. 6). There was no main effect of eye movement (*F* = 1.39, *p* = 0.246) or task instruction (*F* = 2.60, *p* = 0.108). As with later TMS timepoints, RMS of the EMG strongly predicted MEP size (*F* = 23.46, *p* < 0.001). The interaction between eye movement and task instruction was not significant (*F* = 0.018, *p* = 0.894).

One sample tests against baseline revealed that MEPs on View-Follow trials (*t* = 0.32, *p* = 0.746) and Intercept-Follow trials (*t* = 1.77, *p* = 0.078) were not significantly different from baseline. In contrast, View-Fixate trials showed a modest but significant suppression (*t* = −2.22, *p* = 0.028), whereas Intercept-Fixate trials did not significantly differ from baseline (*t* = −0.12, *p* = 0.903). Together, these data suggest that the initial instruction to move (or not move) the hands or eyes may contribute to subtle early changes in CSE.

## Discussion

In this work, we measured TMS, eye-tracking, perceptual judgments, and movement kinematics to probe how gaze behavior and motor intent shape motion perception, interception accuracy, and the state of the motor system. We demonstrate that the intention to intercept, rather than differences in visual motion processing or eye movement strategy, is the primary driver of late-stage facilitation in corticospinal circuits. In contrast, passive observation of a moving target resulted in a timing-independent suppression of motor output, especially when the eyes were fixated. Additionally, smooth pursuit eye movements enhanced temporal accuracy and altered perception of target speed during interception. Together, these results suggest that CSE modulation reflects coordination of perceptual, oculomotor, and motor-preparatory signals for precise interceptive timing.

Performance on the speed discrimination task was generally high across response contexts, consistent with classic findings that motion-sensitive cortical areas accurately track stimulus velocity^42,43^. However, introducing an interceptive goal biased perception: participants systematically underestimated target speed when they anticipated intercepting the moving target compared to when they merely observed its motion. This pattern aligns with recent evidence that perceptual judgments can diverge from the sensorimotor estimates used for interception. Goettker et al. (2019) showed that velocity judgments and interception rely on partially distinct information sources, with interception behavior often reflecting different weighting of motion cues than explicit perceptual reports^44^. Our finding that speed is underestimated in an interceptive context therefore suggests that preparing an action shifts the underlying computations away from purely perceptual velocity estimation and toward the sensorimotor demands of interception. This interpretation is also consistent with embodied-perception theories, which propose that action intentions can bias sensory judgments^45,46^, potentially by engaging an anticipatory motor set that alters how speed cues are processed.

Further, we found that the underestimation of target speed during interception was strongest when participants used smooth pursuit eye movements, indicating that the combination of pursuit and action intent biases perceived target motion^7,47^. One explanation for the differences in speed perception between interception and passive viewing during smooth pursuit is that the timing of catch-up saccades may have differed between tasks, influencing subsequent speed judgments^44^.

In the present study, perceiving the target as moving more slowly may have improved interception timing, consistent with the evidence that foveating the target aids performance^7,48^. This pattern is consistent with work showing that active tracking promotes tighter sensorimotor coupling and predictive pursuit adjustments^3,49^ and further supports the idea that perception of target motion biases interception performance^50^. In sum, our results reveal that motion perception is not a passive readout of retinal input but is dynamically tuned by the brain’s preparatory state.

When participants planned to intercept a moving target, CSE exhibited the expected late-stage facilitation that peaked immediately before movement onset. In contrast, there was no CSE facilitation when passively viewing – instead, CSE remained at baseline (“Follow” blocks) or was suppressed relative to baseline (“Fixation”) blocks. This finding suggests that sensory observation of object motion alone is insufficient to drive corticospinal up-regulation,^51^ consistent with predictive control models of anticipatory visuomotor commands^52,53^.

Contrary to classic reports of an initial inhibitory phase^54,55^, and our previous study^5^, we did not observe a reliable early suppression during interception planning. This lack of an early trough may reflect our dual-task design: while preparing the motor response, participants simultaneously encoded target speed for a subsequent discrimination test. Maintaining readiness for both perceptual and motor demands could elevate baseline excitability or attenuate transient inhibition, effectively masking a brief early suppression. This interpretation aligns with dual-task and attentional-load findings showing that concurrent cognitive-motor demands can reduce intracortical inhibition or increase corticospinal excitability relative to single-task conditions^56–58^. Additionally, our earliest TMS probe at 200 ms post-cue may have missed an earlier inhibitory dip occurring immediately after the instruction cue^59,60^.

We found a limited effect of gaze strategy on corticospinal modulation. Previous work has shown that eye movements can suppress or facilitate corticospinal excitability of the hand muscle, even when the hand is at rest^36,61,62^. In our previous study, eye movements were unconstrained, and participants likely used a combination of smooth pursuits and saccades to track the target^49^. In the present study, we explicitly instructed participants to either fixate on the interception zone or smoothly track the target and used an eye-tracker to monitor eye movements in real-time. Despite differences in interceptive performance, we observed a similar pattern of suppression and late facilitation for both fixation and smooth pursuit conditions during interception. During passive viewing, we found evidence for suppression relative to baseline for the fixation condition only – this may be related to inhibiting eye movements during target motion^63–65^. In contrast, when the oculomotor system is actively engaged, particularly during smooth pursuit, it recruits cortical and subcortical motor networks that overlap with those involved in limb movement planning^66,67^. Functional imaging and electrophysiological studies suggest that pursuit-related activity in frontal and supplementary eye fields can modulate motor cortical output, potentially priming the motor system for coordinated action^8,68^.

In our previous study, we observed evidence of initial suppression as early as 500 ms before movement onset^5^. This led us to wonder whether suppression may be related to processing the task goal itself, rather than only action-specific modulation. Indeed, neuroimaging and electrophysiological studies have shown that top-down task goals can modulate neural activity in sensory and motor regions well in advance of movement, consistent with proactive control mechanisms^69,70^. To address this question, we measured CSE 200 ms after the instruction cue to intercept or view. We found a modest, non-significant trend toward larger MEPs when participants were instructed to intercept versus when instructed to view. Only the Fixate-View condition showed significantly more suppression relative to baseline. This suggests that merely knowing that no eye or hand movement would follow may elicit a tonic down-regulation of corticospinal output^71,72^.

## Conclusion

The present study demonstrates that timed interception of moving targets relies on the coordinated interactions among perceptual judgments, gaze behavior, and preparatory motor signals. Nevertheless, corticospinal facilitation emerges only when an action plan is engaged, indicating that motor intention, rather than continuous visual tracking, ultimately drives corticospinal facilitation. Smooth pursuit sharpened interception timing but did not shift CSE from suppression to facilitation, whereas passive viewing while fixating maintained suppression throughout target motion. In addition, a pre-trial instruction about the task goal alone was insufficient to evoke early facilitation. Complementary speed-discrimination results showed robust encoding of target speed^6^ but underestimation of target speed when participants prepared to intercept – particularly when engaging in smooth pursuit. Together, these findings indicate that gaze aids prediction, but the transition from corticospinal suppression to facilitation is primarily governed by motor intent.

## New & Noteworthy:

This study dissociates the contributions of visual tracking, eye movements, and motor intention to corticospinal excitability during interception. Here, we show that corticospinal facilitation emerges only when an action is planned, whereas passive viewing maintains suppression despite identical visual input. Smooth pursuit enhanced perceptual accuracy and interception timing but had a limited effect on corticospinal excitability, highlighting motor intention as the key driver of preparatory motor output.
